# Description of *Geocenamus vietnamensis* sp. n. (Nematoda: Merliniidae) from Vietnam

**DOI:** 10.21307/jofnem-2019-025

**Published:** 2019-05-27

**Authors:** Huu Tien Nguyen, Thi Mai Linh Le, Thi Duyen Nguyen, Gracia Liebanas, Thi Anh Duong Nguyen, Quang Phap Trinh

**Affiliations:** 1 Institute of Ecology and Biological Resources, Vietnam Academy of Sciences and Technology, 18 Hoang Quoc Viet, Cau Giay, 100000, Hanoi, Vietnam; 2 Graduate University of Science and Technology, Vietnam Academy of Sciences and Technology, 18 Hoang Quoc Viet, Cau Giay, 100000, Hanoi, Vietnam; 3 Departamento de Biología Animal, Biología Vegetal y Ecología, Universidad de Jaén, Campus ‘Las Lagunillas’ s/n, 23071, Jaén, Spain

**Keywords:** Morphology, Molecular, New species, SEM, Taxonomy, D2–D3, ITS, 28S, *Casuarina equisetifolia*, Casuarinaceae, *Amomum aromaticum*, Zingiberaceae

## Abstract

A new species of the genus *Geocenamus* was isolated from soil and root samples from the rhizosphere of *Casuarina equisetifolia* (Casuarinaceae) in Quang Nam province, Vietnam. This species is characterized by a round-to-hexagonal labial disc, the presence of a labial region, which is continuous or slightly offset from the body with six sectors, lateral sectors of first labial annulus being smaller than the submedian sectors, the presence of six to seven labial annules; the absence of deirids; stylet length 24 to 28 μm long, body length 776 to 979 μm long; lateral field with six to eight lateral lines, without areolation at mid-body and with areolation in outer bands at the tail region and a pointed tail terminus. *Geocenamus vietnamensis* n. sp. most closely resembles *G. boghiae* in having a non-sclerotized head framework and lacking a bursa in the males. It can be clearly distinguished from all other species of the genus *Geocenamus* by these characteristics. The combination of morphology, morphometric features, and phylogenetic trees, based on D2–D3 of 28S and ITS rDNA sequences, showed that this new species can be clearly separated from all other sequenced species. This record is the first for *Geocenamus* in Vietnam.

The genus *Geocenamus* was proposed by Thorne and Malek (1968), with *G. tenuidens* (Thorne and Malek, 1968) as its type species. After that Fortuner and Luc (1987) and Maggenti et al. (1988) placed the genus *Geocenamus* in the subfamily Belonolaiminae (Whitehead, 1959). The classifications presented by Siddiqi (2000) distinguished the following five genera within the subfamily Merliniinae: *Geocenamus*, *Nagelus*, *Merlinius* (Siddiqi, 1970), *Scutylenchus*, and *Amplimerlinius* (Siddiqi, 1976). However, Geraert (2011) retained only the genera *Amplimerlinius*, *Geocenamus*, and *Nagelus* in subfamily Merliniinae of the family Dolichodoridae. Remarkably, the main morphological characters that distinguish *Scutylenchus* from other genera in the subfamily Merliniinae are the longitudinal striation of the cuticle and the absence of deirids (Siddiqi, 2000). Deirids are also absent in *Geocenamus* species, but they are obviously present in all other members of the family Merliniidae. Handoo et al. (2007) considered the number of lateral lines as one of the most important characters in identifying these genera that are ranging from three to six lines.

Based on the observations from two studies, Sturhan (2011, 2012) concluded that the absence of longitudinal cuticular striae along the entire body appears to be the only essential character that distinguishes *Geocenamus* from *Scutylenchus*. However, this character has not been considered in classifications of *Tylenchorhynchus* and *Rotylenchus* species (Sturhan, 2012). Therefore, Sturhan (2012) agreed with Brzeski (1991) in synonymizing *Scutylenchus* with the “senior” genus *Geocenamus,* but he considered *Merlinius* as a valid genus.

Following the classification of Sturhan (2012), this study provides morphological and molecular characterizations of a new species of the genus *Geocenamus* in Vietnam.

## Materials and methods

### Nematode population sampling

Soil and root samples were collected from the rhizosphere of *Casuarina equisetifolia* (Casuarinaceae) in Quang Nam province in May 2017. Nematodes were extracted from the soil and the roots by the method described by Nguyen (2003).

### Morphological studies

#### Light microscopy

For morphometric and morphological examination, the extracted nematodes were killed by heat, fixed in a TAF solution, processed to anhydrous glycerol, following Seinhorst (1959), and mounted on permanent glass slides. Nematodes on permanent slides were observed through a Carl Zeiss Axio Lab. A1 light microscope. Measurements and pictures were taken using the ZEN lite software on ZEISS Axiocam ERc5s digital camera. Raw photographs were edited with Adobe Illustrator CS 3.

#### Scanning electron microscopy (SEM)

After examination and identification, good specimens were selected for SEM imaging, following the protocol of Abolafia (2015). The nematodes were hydrated in distilled water, dehydrated in a graded ethanol and acetone series, critical point dried, coated with gold, and observed with a Zeiss Merlin Scanning Electron Microscope.

### Molecular studies

#### DNA extraction, PCR, and sequencing

DNA was extracted from a single individual nematode, following Waeyenberge et al. (2000). The nematode was transferred to a 0.5 ml Eppendorf tube, containing 18 µl of Worm Lysis Buffer (50 mM KCL, 10 mM Tris pH 8.3, 2.5 mMMgCl2, 0.45% NP 40, and 0.45% Tween 20) and 2 µl of proteinase K (600 mg ml^−1^) (Thermo Scientific). The tubes were incubated at 65^o^C (1 hr) and then at 95^o^C (15 min). PCR and sequencing protocols are described in detail by De Ley et al. (1999). Primers for D2–D3 of 28S rDNA amplification were D2A (59-ACAAGTACCGTGGGGAAAGTTG-39) and D3B (59-TCGGAAGGAACCAGCTACTA-39) (Subbotin et al., 2006). Primers for ITS rDNA amplification were modified from Vrain et al. (1992): VRAIN 2F (59-CTTTGTACACACCGCCCGTCGCT-39) and VRAIN 2R (59-TTTCACTCGCCGTTACTAAGGGAATC-39).

#### Phylogenetic analyses

The BLAST homology search program was used to search for closely related species on GenBank. The sequence data set was aligned with the ClustalW software (Thompson et al., 1994). Sequence alignments were manually edited using ChromasPro software (ChromasPro 1.7.4; Technelysium Pty Ltd, Tewantin, QLD, Australia). The sequence data set was analyzed using the maximum likelihood (ML) method in the MEGA 6 program (Tamura et al., 2013). The best fit model for DNA evolution was obtained using the Modeltest in MEGA 6. The model TN93 + G was chosen for D2–D3 of 28S rDNA data set and model GTR + G was chosen for ITS rDNA data set; 1,000 bootstrap replications were executed. Outgroup taxa were chosen according to the results of previously published data (Subbotin et al., 2006; Ghaderi et al., 2014). The trees were visualized in FigTree v1.4.0.

## Results

**Figure 1:**
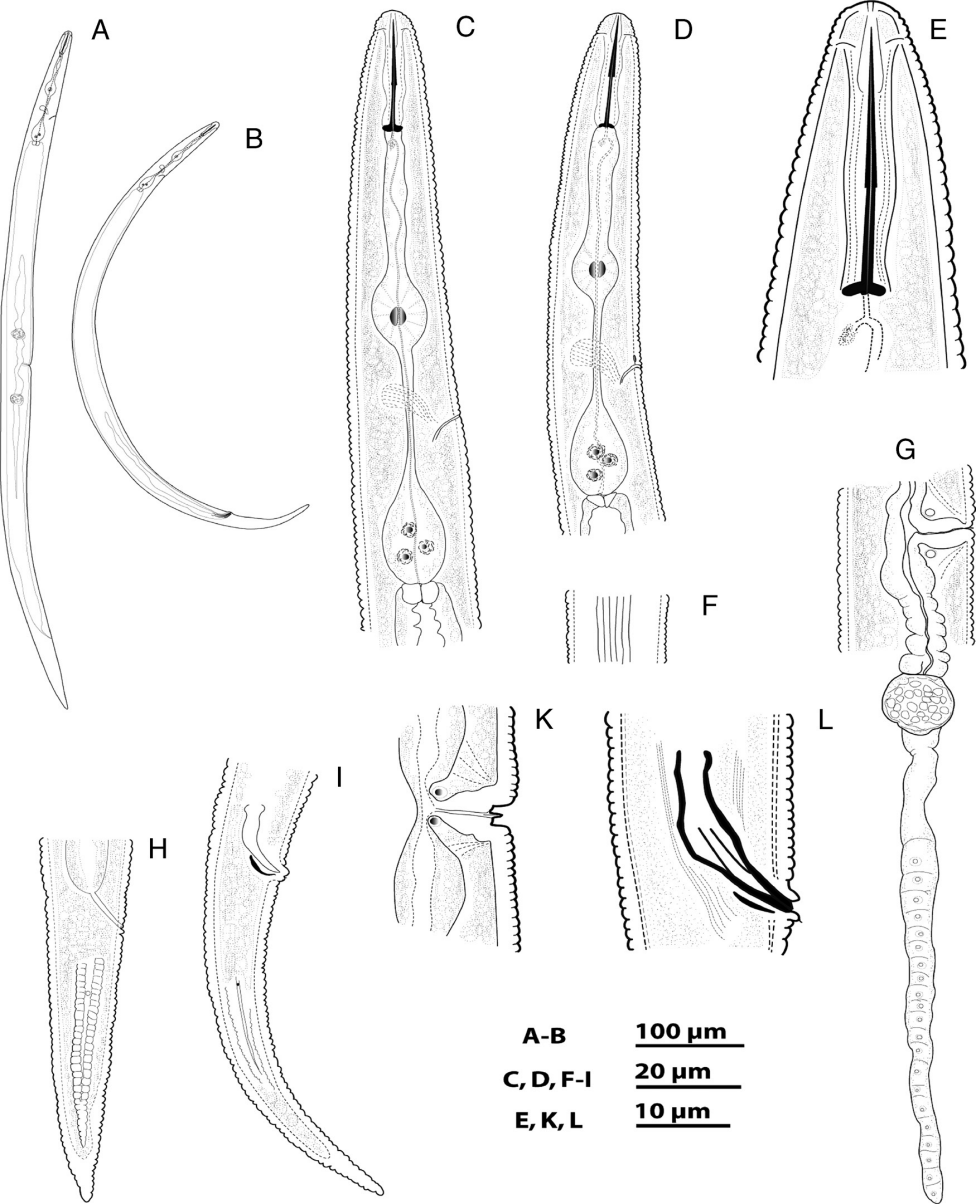
*Geocenamus vietnamensis* n. sp. (♀). (A): Entire body; (C): Pharyngeal region; €: Head region; (F): Lateral field at mid-body; (G): Vulva region with posterior gonad; (H): tail region; (K): Vulva region. (♂). (B): Entire body; (D): Pharyngeal region; (I): Tail region; (L): Cloacal region showing spicule and gubernaculum.

**Figure 2:**
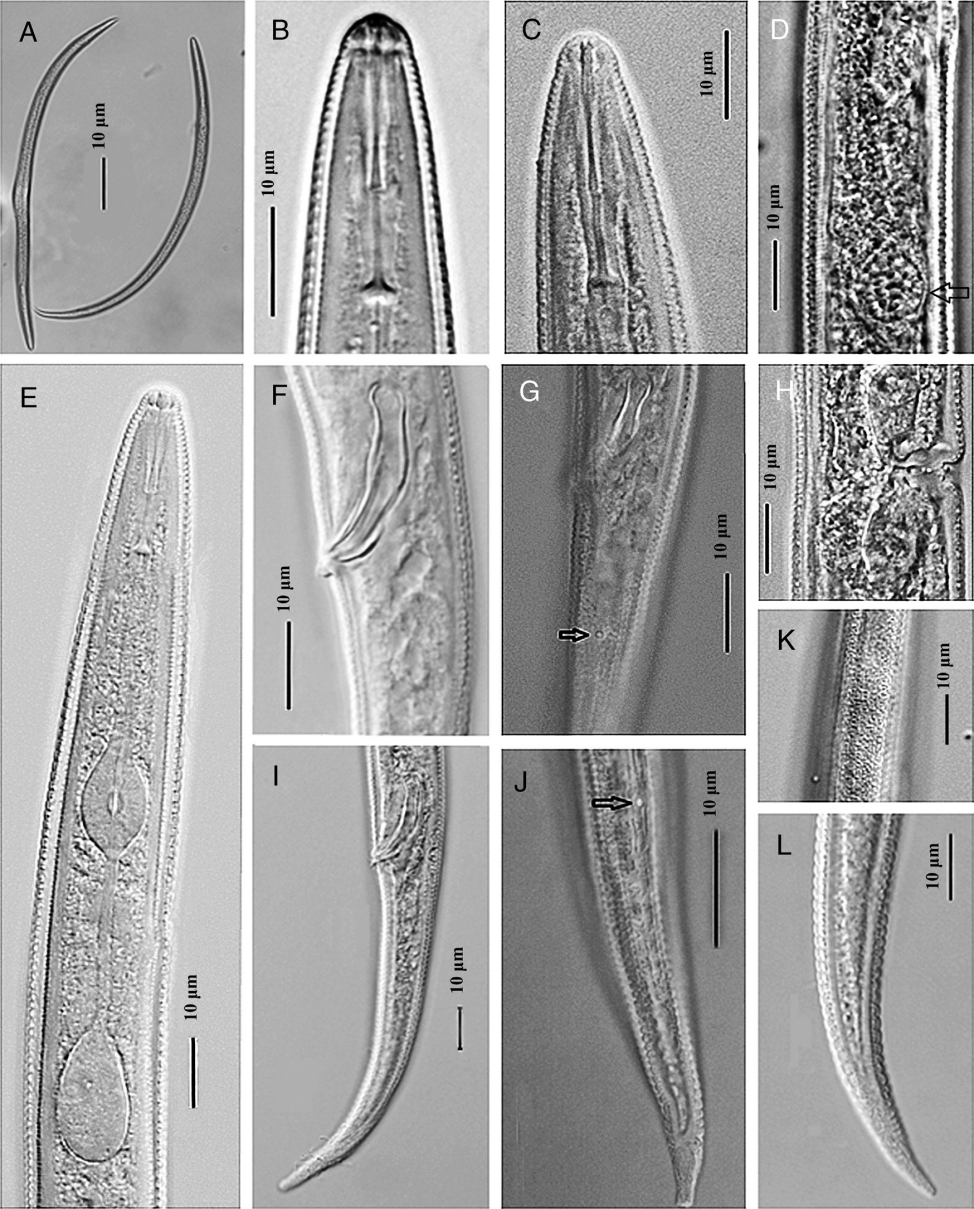
*Geocenamus vietnamensis* n. sp. (Light microscope). (A): Entire body of female and male (female in the left side and male in the right side); (B): Head region of the female; (C): Head region of the male; (D): Vulva region with spermatheca at arrow position; (E): Female pharyngeal region; (F): Spicule and Gubernaculum; (G): Phasmid in the tail of the male; (H): Vulva region; (I): Tail of the male; (J): Phasmid in the tail of the female; (K): Lateral field; (L): Tail of the female.

**Figure 3:**
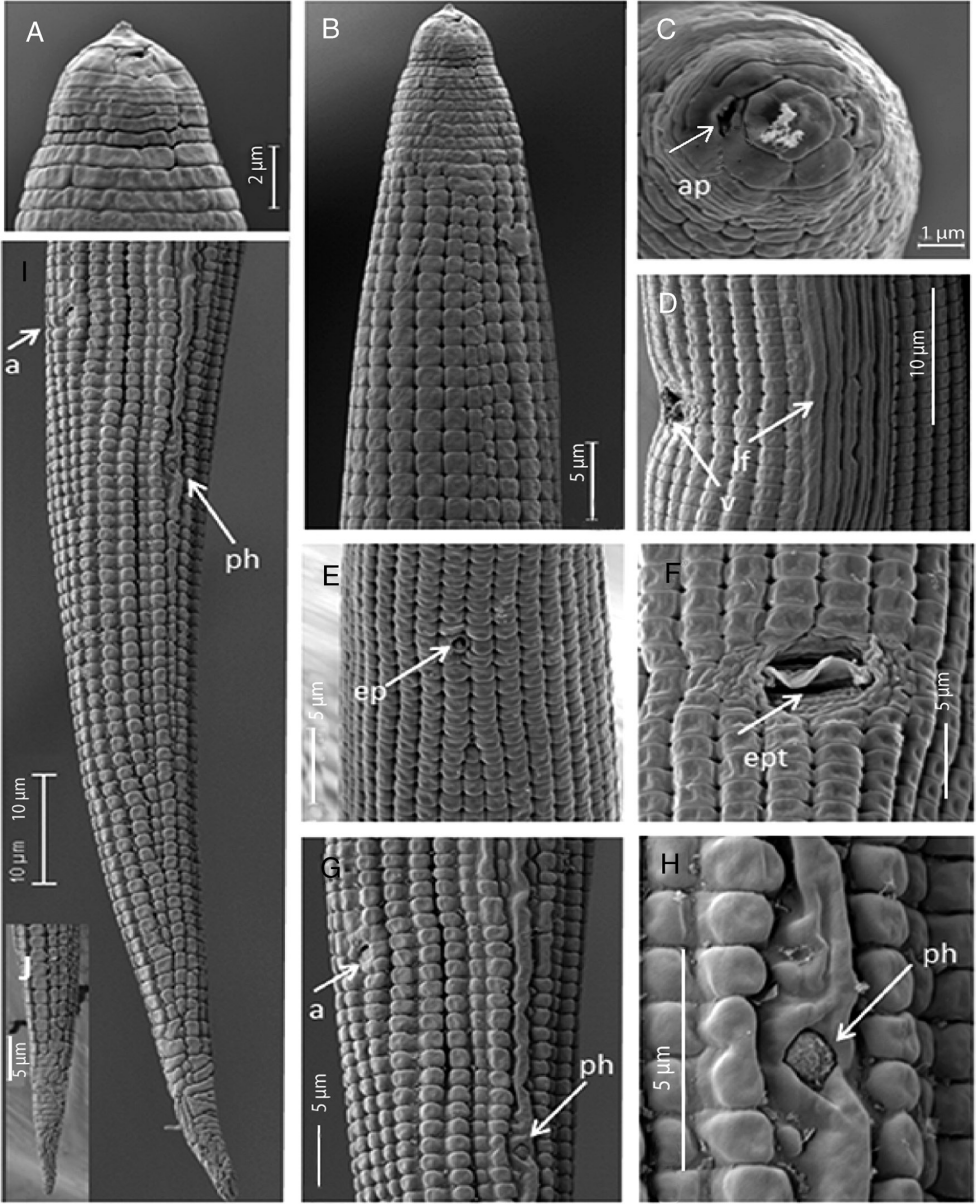
*Geocenamus vietnamensis* n. sp. (SEM ♀). (A): Labial region; (B): Head region; (C): Enface view with amphid orifice (ap) at arrow position; (D): Vulva region with lateral field at arrow position; €: Secretory–excretory pore (ep); (F): Vulva with epyptigma (ept) at arrow position; (G): anus position (a) and phasmid position (ph); (H): phasmid position; (I): Tail region; (J): Tail tip.

**Figure 4:**
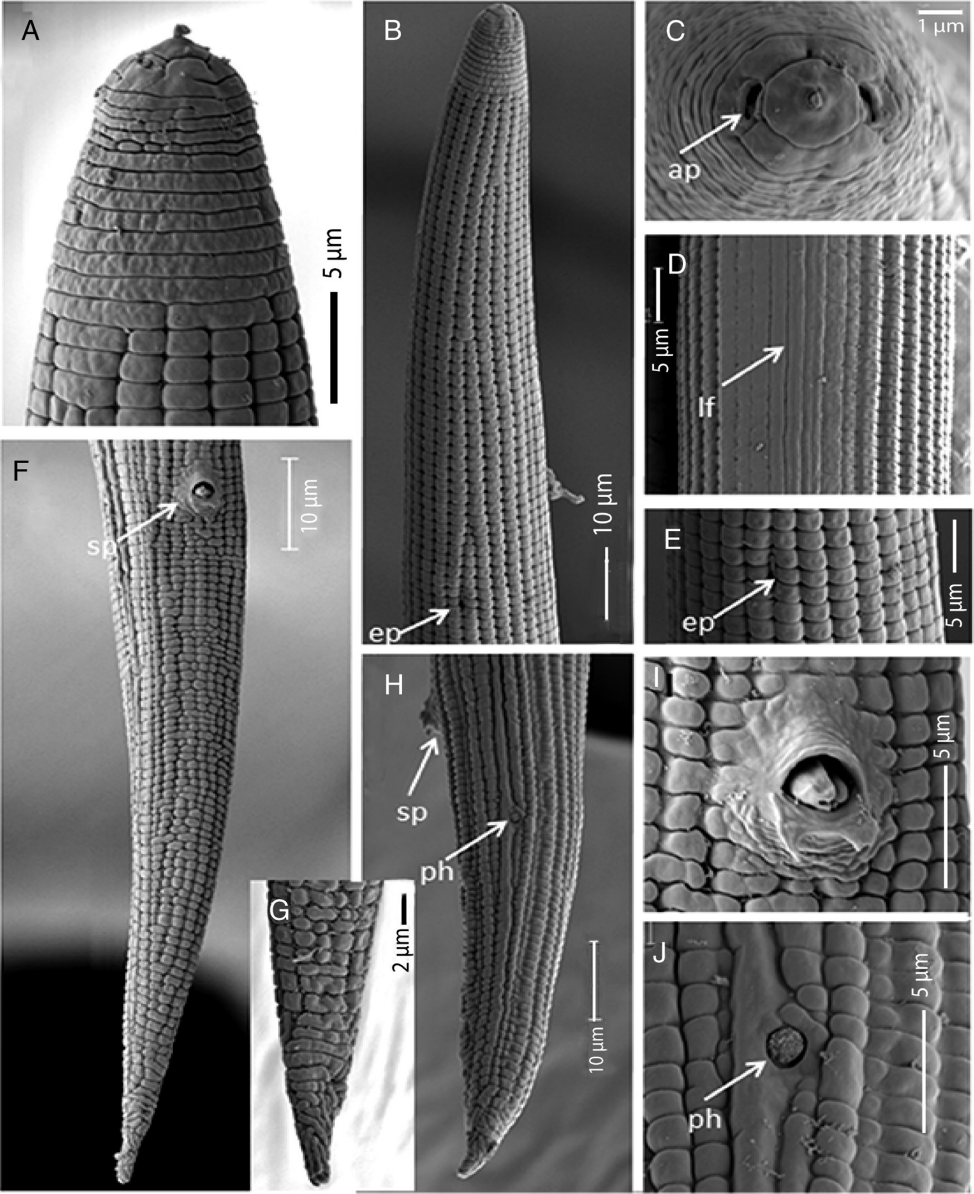
*Geocenamus vietnamensis* n. sp. (SEM ♂). (A): Labial region; (B): Head region; (C): Enface view with amphid orifice (ap) at arrow position; (D): Lateral field; (E): Secretory–excretory pore; (F): Tail region with spicule (sp); (G): Tail tip; (H): Side view of tail with spicule (sp) and phasmid (ph) positions; (I): Cloacal region; (I): Cloaca with hypotigma; (J): Phasmid position.

**Figure 5:**
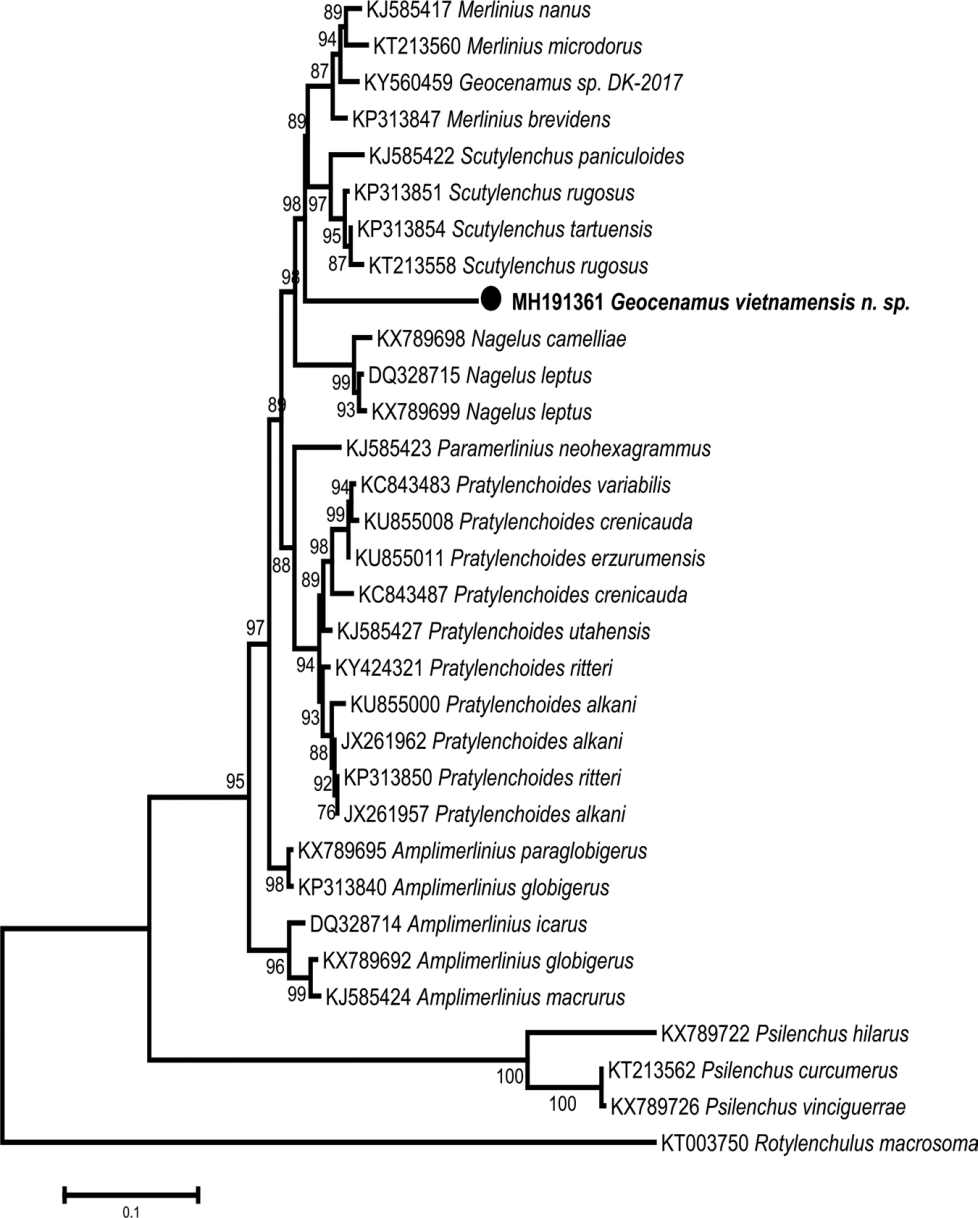
Phylogenetic tree generated from D2–D3 of 28S rDNA sequences based on ML method (TN93 + G model) with 1,000 replications.

**Figure 6:**
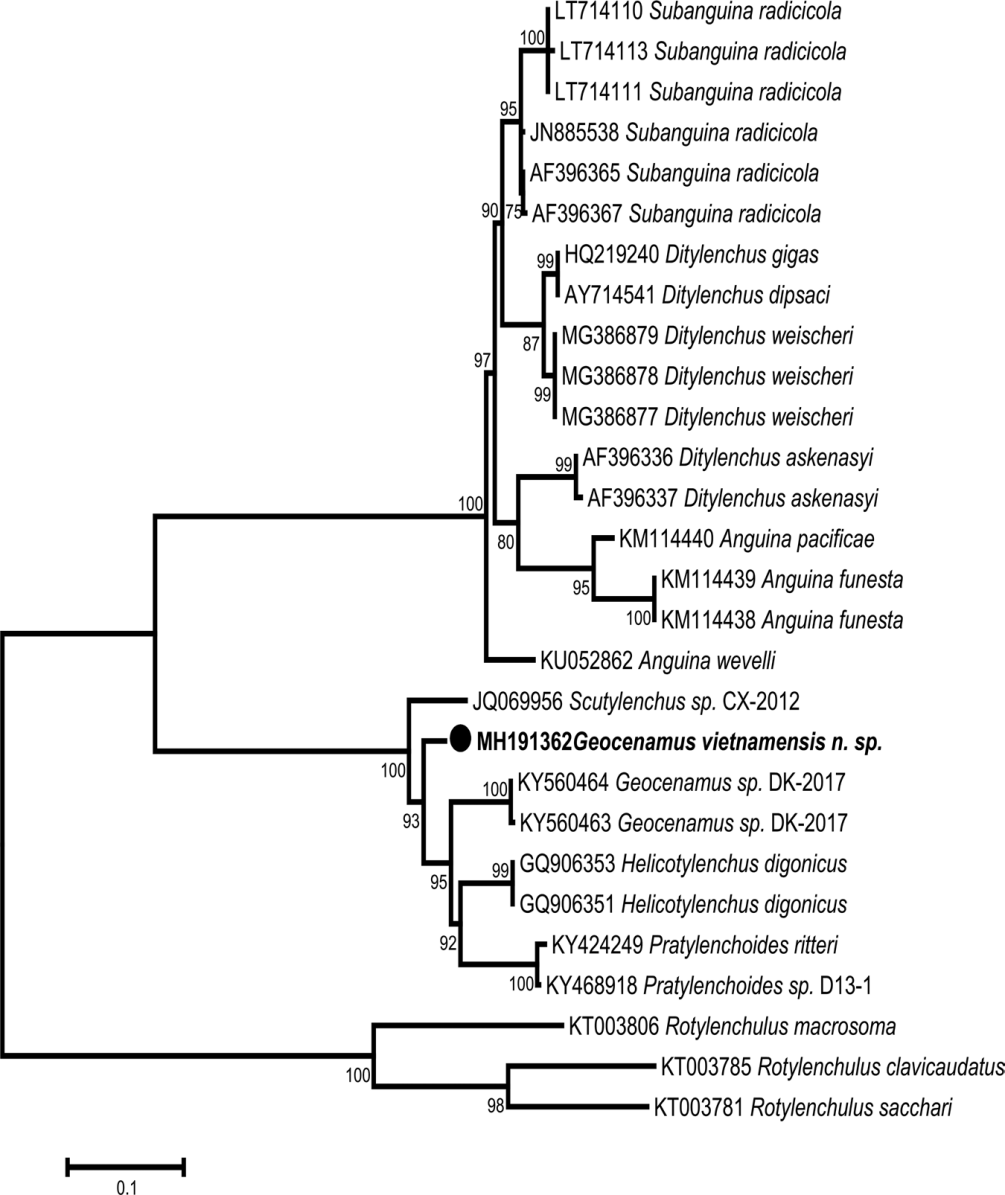
Phylogenetic tree generated from ITS rDNA sequences based on ML method (GTR + G model) with 1,000 replications.

*Geocenamus vietnamensis* n. sp.

(Figs. 1–6, Table 1).

**Table 1.** Morphometric data of *Geocenamus vietnamensis* n. sp. from fixed specimens. All measurements are in μm (except for ratio) and in the form: mean ± s.d. (range).

			Paratypes	
*Geocenamus* measurement	Holotype female	Allotype male	Female (*n* = 18)	Male (*n* = 15)
Body length (L)	877	712	875 ± 64 (776–979)	706 ± 35 (631–754)
Lip region height	3.1	3.6	3.6 ± 0.5 (3.1–4.2)	3.4 ± 0.3 (3.1–3.6)
Lip region width	6.2	5.7	6.5 ± 0.7 (5.2–7.3)	6.1 ± 0.5 (5.2–6.8)
Stylet cone	18.7	15.6	15.7 ± 1.4 (13.5–18.7)	15.1 ± 1.4 (13–17.7)
Stylet shaft	9.4	8.3	10.4 ± 0.5 (9.4–11.4)	8.8 ± 1.6 (5.7–10.9)
Stylet length	28	24	26 ± 1 (24–28)	24 ± 1 (23–26)
Stylet knob height	2.1	1.6	2.0 ± 0.4 (1.6–2.6)	1.9 ± 0.4 (1.6–2.6)
Stylet knob width	4.2	3.1	3.5 ± 0.8 (1.6–4.2)	3.6 ± 0.2 (3.12–3.6)
Stylet base to dorsal gland orifice (DGO)	3.1	3.12	2.9 ± 0.3 (2.6–3.1)	3.0 ± 0.3 (2.6–3.6)
Body width at stylet base	16.6	11.9	16.7 ± 2.8 (13.0–20.8)	13.7 ± 1.0 (11.9–15.6)
Maximum body diameter (MBD)	31	22	29 ± 3 (25–34)	22 ± 1 (22–25)
Body width at vulva	22		27 ± 3 (22–34)	
Body width at anus	18.7	13	19.1 ± 2.4 (15.6–23.9)	16.1 ± 2.6 (13–21.8)
Anterior end to the end of pharynx	152	125	138 ± 9 (124–152)	122 ± 7 (114–135)
Anterior end to the end of median bulb	89	73	81 ± 8 (74–99)	72 ± 5 (67–81)
Anterior end to median valve	78	65	72 ± 7 (66–88)	63 ± 5 (57–71)
Median bulb width	10.9	9.8	11.0 ± 0.7 (10.4–12.5)	9.6 ± 1.2 (6.8–10.4)
Median bulb length	15.6	15.6	17.0 ± 1.6 (14.6–18.7)	15.5 ± 1.2 (13.5–17.7)
Anterior end to nerve ring	86	86	90 ± 6 (83–104)	83 ± 8 (74–99)
Anterior end to secretory–excretory pore	94	89	94 ± 5 (86–102)	86 ± 6 (76–97)
Isthmus length	39	31	32 ± 4 (26–39)	30 ± 2 (26–31)
Basal bulb length	27	21	25 ± 2 (23–28)	22 ± 2 (19–25)
Tail length	86	75	104 ± 14 (85–125)	84 ± 9 (68–96)
Spicule length		21		20 ± 1 (19–22)
Gubernaculum length		5.2		6.5 ± 1.0 (5.2–7.8)
Anus to phasmid	23	17.7	23 ± 3 (18.7–26)	18.6 ± 2.6 (13.5–23)
Tail annuli (ventral)	61	48	64 ± 5 (58–73)	59 ± 6 (48–65)
a = L/MBD	28	33	30 ± 3 (25–34)	32 ± 1 (29–33)
b′ = L/distance from anterior end to pharyngo–intestinal valve	5.8	5.7	6.4 ± 0.5 (5.8–7.2)	5.8 ± 0.2 (5.5–6.2)
c = L/Tail length	10.2	9.5	8.5 ± 0.8 (7.3–10.2)	8.5 ± 0.7 (9.3–9.5)
c′ = Tail length/ABD	4.6	5.8	5.5 ± 0.7 (4.3–6.5)	5.3 ± 0.7 (4.3–6.3)
V = Anterior end to vulva/L ×100	50		49 ± 2 (47–52)	
M = stylet conus length/stylet length ×100	67	65	60 ± 3 (56–67)	62 ± 4 (57–69)
MB = % distance from anterior end to median bulb in relation to the length of pharynx	51	52	52 ± 3 (49–59)	51 ± 1 (50–53)
O = % distance of dorsal pharyngeal gland opening from stylet knobs in relation to the stylet length	11.1	13.0	11.0 ± 1.3 (9.6–13.0)	12.5 ± 1.3 (10.2–14.3)
Phasmid % tail	27	24	22 ± 2 (18.3–27)	22 ± 3 (17–28)

### Description

#### Female

Their body is ventrally curved after fixation, tapered at both ends. The lateral field is present with 6 to 8 lines without areolation at mid-body, about 6.0 µm wide (Figs. 1F, 2K, 3D). Body annuli are distinct, 1.2 ± 0.1 µm wide, and they are divided into blocks. The longitudinal striation is conspicuous with 18 to 20 lines at the ventral side near vulva (Fig. 3D). The labial region bears 6 to 7 annuli without longitudinal striae, not offset or very slightly offset by a shallow depression. A projected, round-to-hexagonal oral disc is present. First labial annulus is divided into six sectors; the lateral sectors are much smaller than the other four. Lateral sectors of first annulus are present with oval amphidial apertures (Fig. 3C). The head framework is not sclerotized. A well-developed stylet is present with elongated conical part and sloping and rounded knobs (Figs. 1C,E, 2B,E). The dorsal gland orifice is located 2.6 to 3.1 µm posterior to stylet knobs. An oval median bulb and a saccate terminal bulb, not overlapping intestine, are present. Secretory–excretory pore is located at the level of the nerve ring, 86 to 102 µm from the anterior end; hemizonid is located anterior to the secretory–excretory pore; deirid is absent. Vulva is a transverse slit, sunken or flush to body surface; epyptigma is double; vaginal length is 30% of the body diameter, moderately sclerotized; spermatheca is rounded, with rounded sperm cells; ovaries are outstretched with a single row of oocytes. The tail is conical, annulated with hyaline, and the tail tip is pointed; 58 to 73 tail annuli are present on the ventral side (Figs. 1H, 2L, 3I,J). Phasmid is slightly enlarged, at about one-fourth of the tail length. The lateral field ends before the hyaline part of the tail tip.

#### Male

Males are similar to females but they are more ventrally arcuate (Figs. 1B, 2A). The male reproductive apparatus is present with slightly bent spicules and a curved gubernaculum; cloacal lips are protuberant. Two posterior hypoptygmata are well developed, with equal lengths (Fig. 4I). Bursa is absent. Phasmid is located 22 to 28 µm posterior to the cloacal aperture. The tail is conical, annulated with hyaline, and the tail tip is pointed (Figs. 1I, 2I, 4F,G,H).

### Molecular characteristics

#### D2–D3 of 28S rDNA

The alignment of D2–D3 of 28S rDNA sequences contained 31 sequences including the sequences from two outgroup taxa (*Psilenchus* spp. and *Rotylenchulus macrosoma*). The length of alignment was 602 bp. The D2–D3 of 28S rDNA sequence of *Geocenamus vietnamensis* n. sp. is 91 to 93% similar to those of *Merlinius* spp. (KP313847, KT213560, KJ585417), *Scutylenchus* spp. (KT213558, KP313854, KP313851, KJ585422), and *Geocenamus* sp. (KY560459). The variations between the D2–D3 of 28S rDNA sequence of *G. vietnamensis* n. sp. and other studied species were 10% (70 bp) compared to the sequence of *Geocenamus* sp. (KY560459), 10 to 13% (68-91 bp), 10 to 11% (68–78 bp) compared to the sequences of *Merlinius* spp., 10 to 12% (69–82 bp) compared to the sequences of *Scutylenchus* spp., 11 to 12% (77–79 bp) compared to the sequences of *Nagelus* spp., and 12 to 13% (81–91 bp) compared to the sequences of *Pratylenchoides* spp. The variations were 26 to 28% (186–196 bp) compared to two outgroup taxa (*Psilenchus* and *Rotylenchulus*).

The phylogenetic relationships between *G. vietnamensis* n. sp. and other species, based on the maximum likelihood method (GTR+G model), were presented in Figure 5. The D2–D3 of 28S rDNA sequence of *Geocenamus vietnamensis* n. sp. has a sister relationship with *Geocenamus* sp. (KY560459), *Scutylenchus* spp. (KT213558, KP313854, KP313851, KJ585422), and *Merlinius* spp. (KP313847, KT213560, KJ585417). Together they form a group with 98% bootstrap support.

#### ITS rDNA

The ITS rDNA sequence alignment contained 27 sequences including the sequences from *Rotylenchulus* spp. (outgroup taxon). The length of the alignment was 897 bp. The ITS rDNA sequence of *Geocenamus vietnamensis* n. sp. is 88 to 91% similar to those of *Geocenamus* sp (KY560464, KY560463), *Scutylenchus* sp (JQ069956), *Helicotylenchus digonicus* (GQ906353, GQ906351), and *Pratylenchoides* spp. (KY424249, KY468918). The sequence variations between the ITS rDNA sequence of *G. vietnamensis* n. sp. and other species varied from 6 to 10% (63–99 bp) compared to *Geocenamus* sp. (KY560464, KY560463), 6% (58 bp) compared to *Scutylenchus* sp. (JQ069956), 6% (61 bp) compared to *Helicotylenchus digonicus* (GQ906353, GQ906351), 85 to 86% compared to *Pratylenchoides* spp. (KY424249, KY468918), and 30 to 35% (264-318 bp) compared to outgroup taxa (KT003781, KT003785, KT003806).

The phylogenetic relationships between the ITS rDNA sequence of *Geocenamus vietnamensis* n. sp. and other species, based on maximum likelihood (TN92 + G model), are presented in Figure 6. The ITS rDNA sequence of *G. vietnamensis* n. sp. has a sister relationship with the sequences of *Geocenamus* sp. (KY560464, KY560463), *Scutylenchus* sp (JQ069956), *Helicotylenchus digonicus* (GQ906353, GQ906351), and *Pratylenchoides* spp. (KY424249, KY468918). They were placed together in a clade with maximal bootstrap support (100%).

### Diagnosis

*Geocenamus vietnamensis* n. sp. is characterized by a long stylet, a slightly offset labial region, a non-sclerotized head framework, a labial region bearing 6 to 7 annuli without striation, body annuli divided into blocks, a conical and pointed tail with annulated, hyaline tip, and the absence of a bursa in the males.

### Relationships

Among *Geocenamus* spp., *G. vietnamensis* n. sp. is most similar to *G. boghiae* (Choi and Geraert, 1993) in having a non-sclerotized head framework and the males without a bursa. They can be differentiated from all other *Geocenamus* spp. by these characteristics. However, this new species differs from *G. boghiae* in having a shorter stylet (24–28 vs 48–54 μm and 22.9–25.5 vs 45–52 μm in females and males, respectively), a shorter body length in males (631–754 vs 750–900 μm), a shorter length of the anterior end to the end of pharynx (124–152 vs 162–185 μm and 114–135 vs 134–167 μm in females and males, respectively), a longer female tail (85–125 vs 52–69 μm), a shorter spicule length (18.7–22 vs 22.35 μm), a shorter gubernaculum length (5.2–7.8 vs 9–11 μm), a more anterior vulva (47–52 vs 50–57), a smaller c value (7.3–10.2 vs 14.2–18.3 and 9.3–9.5 vs 10.9–15.5 in females and males, respectively), and a larger c value (4.3–6.5 vs 2–3.2 and 4.3–6.3 vs 2.7–3.8 in females and males, respectively).

### Type host and locality

One holotype female, one allotype male and 33 paratypes (18 females and 15 males) from a population were extracted from the rhizosphere of *Casuarina equisetifolia* (Australian pine tree) (Casuarinaceae) in Quang Nam Province, Vietnam (15°56′56′′ N, 108°30′36′′ E).

### Etymology

The specific epithet is derived from Vietnam, the country where the species was found.

### Type material

Holotype and paratypes were deposited in the Nematode Collection of the Institute of Ecology and Biological Resources (IEBR), Vietnamese Academy of Science and Technology, 18 Hoang Quoc Viet Road, Hanoi, Vietnam, and 10 female paratypes were deposited in the nematode collection of the Zoology Museum, Ghent University, K. L. Ledeganckstraat 35, Ghent, Belgium. The D2–D3 and ITS sequences were deposited in GenBank with accession numbers MH191361 and MH191362, respectively.

## Discussion

In terms of morphology, this new species possesses a relatively long stylet compared to other plant-parasitic nematode groups, a slightly offset labial region bearing 6 to 7 annuli without striation, a non-sclerotized head framework, a body cuticle with 26 to 28 longitudinal striae (excluding lateral lines), 6 to 8 lateral lines at lateral field, a conical and pointed tail with annulated tip, and the absence of a bursa in the male. These characteristics clearly indicate that this species belongs to the subfamily Merliniinae. According to Siddiqi (2000) and Sturhan (2011), this species should belong to the genus *Scutylenchus* due to the appearance of tessellated cuticle and the absence of deirids. However, by considering the inconsistency in the appearance of longitudinal striae in *Rotylenchus* and *Tylenchorhynchus*, Sturhan (2012) agreed with Brzeski (1991) to synonymize *Scutylenchus* with *Geocenamus*. Interestingly, although the classification of Geraert (2011) is in agreement with Sturhan (2012), he considered the absence of radial grooves on the labial region as the key feature to define the genus *Nagelus* in the subfamily Merliniinae. It is a bit confusing in classifying our species due to the absence of radial grooves on the labial region, but this species can be clearly excluded from *Nagelus* because of the absence of deirids. In conclusion, we placed this new species in the genus *Geocenamus* according to the most recent classification.

In both D2–D3 of 28S and ITS rDNA trees, our new species always shows a very close relationship with species of *Scutylenchus* and *Geocenamus*, and a more distant relationship with *Nagelus*. In this study, the sister relationship of the genera *Merlinius*, *Scutylenchus*, and *Geocenamus* in the ITS rDNA tree strongly supports the recent classification in the subfamily Merliniinae. However, the number of D2–D3 of 28S and ITS rDNA sequences in GenBank for Merliniinae, as well as the genus *Geocenamus*, are very limited. More molecular data are needed to further resolve relationships in Merliniinae.
